# Attitudes towards the ‘Shisha No Thanks’ campaign video: Content analysis of Facebook comments

**DOI:** 10.18332/tid/153543

**Published:** 2022-10-18

**Authors:** Lilian Chan, Ben Harris-Roxas, Becky Freeman, Ross MacKenzie, Lisa Woodland, Blythe J. O’Hara

**Affiliations:** 1 Prevention Research Collaboration, Charles Perkins Centre, University of Sydney, Camperdown, Australia; 2 School of Population Health, University of New South Wales, Sydney, Australia; 3 Centre for Primary Health Care and Equity, University of New South Wales, Sydney, Australia; 4 New South Wales Multicultural Health Communication Service, Sydney, Australia

**Keywords:** campaign, shisha, waterpipe, content analysis, social media

## Abstract

**INTRODUCTION:**

While social media are commonly used in public health campaigns, there is a gap in our understanding of what happens after the campaign is seen by the target audience. This study aims to understand how the *Shisha No Thanks* campaign video was received by the Facebook audience by analyzing Facebook comments posted to it. Specifically, this study aims to determine whether the Facebook audience accepted or rejected the campaign’s message.

**METHODS:**

A sample of the Facebook comments was extracted, and the study team, which included cultural support workers, developed content categories consistent with the research question. Each comment was then coded by three team members, and only assigned a category if there was agreement by at least two members.

**RESULTS:**

Of the 4990 comments that were sampled, 9.1% (456) accepted the campaign message, 22.9% (1144) rejected the message, 21.8% (1089) were unclear, and 46.1% (2301) contained only tagged names. Of the sample, 2.8% (138) indicated the commenter took on board the campaign message by expressing an intention to stop smoking shisha, or asking a friend to stop smoking shisha. Of the comments that showed rejection of the campaign, the majority were people dismissing the campaign by laughing at it or expressing pro-shisha sentiments.

**CONCLUSIONS:**

This study demonstrates that conducting content analyses of social media comments can provide important insight into how a campaign message is received by a social media audience.

## INTRODUCTION

While social media have become a ubiquitous channel for public health campaigns, many campaigns primarily use them as one-way broadcast media and measure the effectiveness of their efforts through metrics such as reach and engagement^1^. More comprehensive campaign evaluations also assess summative (impact and outcome) evaluation measures, such as changes in knowledge or behaviors linked to the health message disseminated through social media^2-4^.

Understanding how a public health campaign can change the awareness and attitudes of its intended audience and potentially convince them to reconsider their behaviors, however, requires further analysis^5^. Social media comment analysis has been widely used in health research to understand how the public discusses tobacco and nicotine use^6-9^ and specifically shisha, a form of tobacco smoking^10-12^. Social media comment analysis can provide insight into people’s attitudes in a more informal setting than focus groups or survey responses.

Social media comment analysis has also been used to understand public responses to health campaigns related to tobacco and nicotine use^13^. Largely, this research has been conducted on Twitter content, rather than Facebook which has more restricted access to exporting comments for analysis. To date, no social media comment analysis has been conducted to understand the response to a health campaign about shisha smoking.

### The *Shisha No Thanks* project

Shisha (also known as waterpipe, hookah, narghile or arghile) has been practiced in Arabic-speaking countries for many decades, and the practice is becoming more popular among young people, particularly in Middle Eastern countries^14^. It is also a global trend, spreading to other countries, such as the US and Australia^15-17^. There are many factors contributing to this trend, including the introduction of flavored shisha tobacco, lax regulation of shisha smoking^18^, misconception that shisha smoking is safer than other forms of tobacco smoking^19^, that shisha smoking is cool or fashionable^19-21^, and because it is a social activity^21,22^ with cultural elements^19-23^.

This growing trend is of great concern, as shisha smoking is associated with a range of health harms, including increased risks of esophageal and lung cancer, emphysema and cardiovascular disease^24,25^. Concerningly, among young people, shisha smoking is also associated with double the risk of later initiation of cigarette smoking^26^.

**Figure 1:**
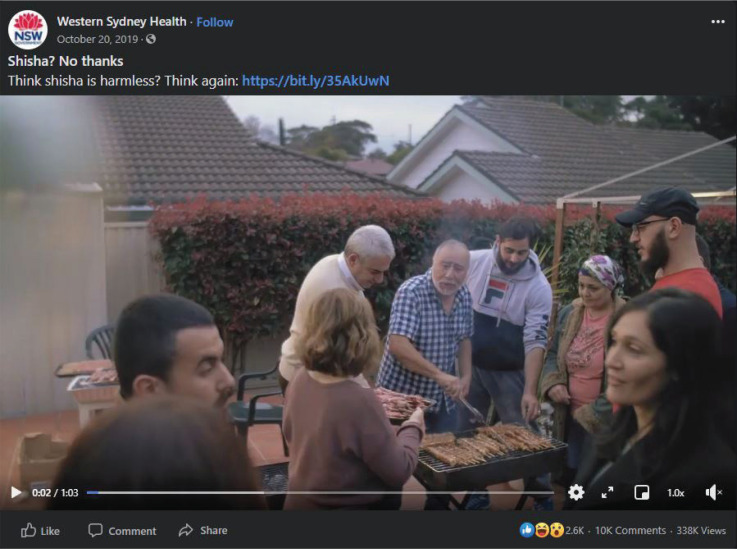
‘Shisha No Thanks’ video on Western Sydney Local Health District’s Facebook page

In response to the situation in Australia, the *Shisha No Thanks* project was pioneered to raise awareness of the harms of shisha smoking among young people (aged 18–35 years) from Arabic-speaking backgrounds in Sydney, New South Wales. In the geographical area of the project, 12% of the population identify themselves as Arabic-speaking^27^, and among Arabic speakers in Sydney, 11.4% reported using shisha^17^. The key objective of the project was to increase community awareness of the harms of shisha smoking. The project took a co-design approach and developed a suite of evidence-based, culturally appropriate campaign resources in both English and Arabic that conveyed the harms of shisha smoking, including factsheets and social media content, which were distributed through community events, public relations activities, and social media (Facebook, Instagram and YouTube). One of the key campaign resources was a 1-min broadcast quality campaign video in English, developed for online viewing, which depicts a scenario of a gathering of family and friends during which shisha is offered to the main character. However, instead of the usual shisha, the head of the shisha was filled with cigarettes and followed by the comment ‘45 minutes of shisha is equivalent to 100 cigarettes’^28^. The video was published on the campaign’s YouTube, Facebook, Instagram and website, as well as shared on a number of Facebook pages of partner organizations, including local health services and community organizations. Western Sydney Local Health District (WSLHD), which is responsible for the delivery of health services in the western suburbs of the city, was one project partner who organically (unpaid) shared the campaign video on its public Facebook page in October 2019 (Figure 1)^29^. The campaign video on WSLHD’s Facebook page received over 10000 comments posted to the video within one week of launching the video. This was a large response in comparison to the number of responses on the other Facebook pages which shared the campaign video (where the number of comments ranged 0–284).

This study analyzes the Facebook comments posted to the *Shisha No Thanks* campaign video to examine how it was received by WSLHD’s Facebook audience. Facebook has been chosen as the social media platform of focus for this study as it was one of the main social media channels used by the *Shisha No Thanks* campaign, and the platform on which there was the most engagement with the campaign. This study aims to address the question of whether the Facebook audience that saw the campaign video accepted the campaign message (i.e. perceived the message as relevant or important), or rejected the message (i.e. dismissed it, did not believe it, or ridiculed it). This research study was conducted in parallel with the impact evaluation of the *Shisha No Thanks* campaign which comprised a pre-post survey asking people about their attitudes about the harms of shisha smoking^30^.

## METHODS

A sample of 5000 Facebook comments on the campaign video post were extracted using Facebook’s Graph API (the platform’s interface which allows extraction of text-based data), with the permission and cooperation of WSLHD. The maximum number of comments that can be exported using Facebook’s Graph API is 5000, and Facebook does not provide public information about how the Graph API samples these comments (e.g. whether by recency or whether it is a random sample). Comments were extracted with the accompanying information of the time the comment was posted, and an ID number of the Facebook user who posted it. The names of people who posted the comments were not extracted. Any names ‘tagged’ (mentioned) in the comments were then manually de-identified. As the exported file displayed emojis as unicode strings (e.g. U+1F600), they were then converted into the emoji image along with the official Common Locale Data Repository Short Name (e.g. 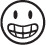 <grinning face>)^31^.

The methodology of this study drew upon the process used by Krauss et al.^12^. After initial familiarization with the data through review of the first 300 comments, we developed content coding categories consistent with the research questions. The three overarching categories of ‘Accept’ the campaign video message, ‘Reject’ the message, and ‘Unclear’, were developed. Common themes were then identified for each of the categories, making up the subcategories for each main category (Table 1).

**Table 1:** Comment categories and subcategories used for coding the data

*Category*	*Subcategory*	*Description*
**Accept**	Intention to stop smoking/asks friend to stop smoking	Comment shows concern for a friend/family member, tells them not to smoke shisha; or that the commenter will think twice before smoking shisha again, or a desire to quit/reduce shisha use
	Agreement with message	Commenter seems to agree with the campaign message (e.g. repeating info from the message), says how important this information is, or shows shock or surprise at the facts
	Other	Other comment that shows acceptance of the campaign video, but does not fit in above categories
**Reject**	Dismiss	Commenter dismisses the message (does not take it seriously) – laughing at it, brushing it off, ridiculing it, or saying that shisha is good/they want to smoke shisha
	Skeptical	Does not believe the message or trust the messenger
	Other	Other comment that shows rejection of campaign video, but does not fit in above categories
**Unclear**	Comment only contains the phrase ‘No thanks’	Comment only contains ‘No thanks’, with/without tagged name(s), with nothing else to indicate the meaning/tone of these comments
	Genuine question	Comment is a genuine question about the facts, suggesting the person wants to know more
	Personal or cultural attack	Commenter feels personally attacked, or suggests they think the video is stereotyping/racist towards a certain group; but does not disregard the message
	Relevant, but meaning unclear	Comment is clearly relevant to the video, but the meaning of the comment is unclear
	Irrelevant or other	Comments that do not make sense, or are irrelevant to the campaign message

The content coding categories were then tested by cultural support workers, who are bilingual health workers employed to work directly with culturally and linguistically diverse communities^32^. The four cultural support workers chosen for this study were in the target audience age group (18–35 years), and two were Arabic-speaking. Their involvement ensured that cultural meanings of the comments (both the culture of young people, and of Arabic-speaking communities) were captured in the content coding process. The cultural support workers provided feedback on whether they felt the content coding categories captured the meaning of the comments correctly, and the categories were modified based on their feedback.

The revised content categories were then tested by the coding team, which was made up of two researchers, the *Shisha No Thanks* project officer, one staff member from WSLHD, and four cultural support workers. The coding team was trained in content analysis and familiarization with the content categories. Instead of estimating inter-coder reliability through coding a small sample of comments, to best ensure consistency of coding, each comment was coded by three coders, with the final coding requiring agreement amongst at least two coders. This ensured that a rigorous coding methodology was used. If there was no agreement between at least two coders for the comment’s category or subcategory, the comment was reviewed by two researchers who discussed which category and subcategory were most appropriate. Once all comments had been assigned a category and subcategory, the number of comments in each category and subcategory were quantified.

Finally, process evaluation metrics, including reach, video views, likes, shares and comments were obtained from WSLHD’s team using Facebook Insights, the platform’s native analytics dashboard.

## RESULTS

The unpaid campaign video post on Western Sydney Local Health District’s Facebook page reached 435811 people, had 316611 3-second video views, and 77351 1-minute video views (24.4% of 3-s video views). As videos play automatically on Facebook, 3-second video views correspond to people who did not immediately scroll past the video and watched at least 3 seconds of the video. After 3 seconds they may have subsequently continued to scroll past it, clicked the stop button, or continued watching more of the video. Similarly, 1-minute video views correspond to people who stayed and watched at least 1 minute of the video, noting that the entire video is only 1:03 min in length. The post garnered over 23470 engagements, which included 1772 shares, and over 11000 comments.

In total, 4991 comments were extracted from the Facebook post using the Facebook Graph API. Of these comments, one comment posted by WSLHD responding to the comments in general was excluded. Of the remaining 4990 comments, 2301 (46.1%) contained only tagged names of other Facebook users, with no other words, 456 (9.1%) accepted the campaign message, 1144 (22.9%) rejected the campaign message, and 1089 (21.8%) were unclear whether they accepted or rejected the campaign message (Table 2).

**Table 2:** Number of comments assigned to each category and subcategory (N=4990)

*Category*	*Subcategory*	*n*	%*
**Accept**	Intention to stop smoking/asks friend to stop smoking	138	2.8
	Agreement with message	278	5.6
	Other	40	0.8
**Reject**	Subtotal	456	9.1
	Dismiss	1010	20.2
	Skeptical	124	2.5
	Other	10	0.2
	Subtotal	1144	22.9
**Unclear**	Comment only contains phrase ‘No thanks’	71	1.4
	Genuine question	17	0.3
	Personal or cultural attack	35	0.7
	Relevant, but meaning unclear	742	14.9
	Irrelevant or other	224	4.5
	Subtotal	1089	21.8
**Names only**		2301	46.1
**Total**		4990	100

* Percent of all comments.

### Accepting the campaign message

*Stop smoking shisha*

Of the 456 comments which were categorized as ‘accepting the campaign message’, 138 (2.8% of comments) included a ‘stop smoking’ idea, which could either be the commenter stating they would no longer smoke shisha, for example:

- Omg I am done [name] [name]
- [name] [name] brb just quitting telling their friend to stop smoking shisha, for example:
- [name] lay off the shish bruv
- Enough is enough [name]
- [name] I love you too much to watch you slowly die at the hands of sisha (*sic*). Pls stahp (*sic*) boo! If not for you, for me!

or that the group should stop smoking shisha, for example:

- [name] [name] yeah alright lets give it a miss 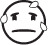
<downcast face with sweat>
- [name][name] no more Granville for us [*Granville is a suburb in Sydney that has shisha bars/lounges*]

The statements varied in intensity, from begging their friend to stop (e.g. ‘pls, cmon it must stop’), to threats (e.g. ‘[name] I'm throwing yours away’), to soft requests (e.g. ‘think again’, ‘be careful’, ‘you need to take it easy’). There were also references to ‘I told you’, suggesting that the commenter had had conversations with their friend previously.

*Agreement with campaign message*

The majority of ‘accept’ comments were subcategorized as ‘agreeing with campaign message’ (n=278; 5.6% of comments). These generally suggested that the commenter had believed and taken on board the campaign message, but did not necessarily indicate any intended behavior change. Types of comments that fit into this category included those that expressed shock or surprise at the campaign facts, for example:

- [ name] holy moly
- [name] 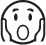
<face screaming in fear> repeating key campaign messages or facts, for example:
- [name] [name] 45 mins = 100 ciggies 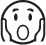
<face screaming in fear>

telling their friend about the campaign message, for example:

- [name] get woke cuz
- [name] [name] wtf do I keep sayingggg (*sic*)
- [name] this is why you should listen to me 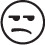
<unamused face>

or showing support for the campaign message, for example:

- Thank goodness this is getting some publicity
- About time for this info. The number of people that have shisha is a joke and worse think it’s harmless.

*Other*

There was a small proportion of ‘agree’ comments which were classified as ‘other’ (n=40; 0.8%), and these generally suggested that the commenter believed the campaign message, but did not intend to change behavior, for example:

- [name] [name] still does it anyways 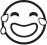
<face with tears of joy>
- [name] for all you shisha lovers.

### Rejecting the campaign message

*Dismissive of campaign message*

Of all subcategories, the ‘dismissive’ subcategory had the largest number of comments (n=1010; 20.2%). These mainly consisted of comments of people laughing at the campaign message/video, for example:

- [name]
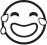
<face with tears of joy>
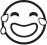
<face with tears of joy>
- [name] omgggg HAHAHA
- [name] I’ve never laughed so much in my life (*sic*)

or comments of people expressing pro-shisha attitudes or behaviors, for example:

- [name] Cbf* <3 shisha [*Cbf denotes a slang euphemism for being too lazy*]
- [name] get me the argilee (sic) cuzz

Some comments in this category were also sarcastic in nature, for example:

- lol this really convinced me to stop wow 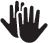
<clapping hands> 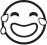
<face with tears of joy>
- [name] [name] does it count as a serving of fruit tho? 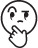
<thinking face>
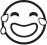
<face with tears of joy> or ridiculing the health harms, for example:
- Rip lungs 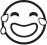
<face with tears of joy>.

*Skeptical about the campaign*

There was also a proportion of comments that suggested skepticism towards the campaign message (n=124; 2.5%). These either said the campaign facts were not true, for example:

- [name] whaaat (*sic*) fake news
- [name] never seen something so inaccurate in my life

or they expressed cynicism about the motivation for the campaign, i.e. that the government makes a lot more money from cigarette tax, so they want people to smoke cigarettes instead of shisha, for example:

- Cigarette tax revenue must be down
- Smoke cigarettes please, we make more tax on those
- Wat a bull…t ad. Only cos there is ZERO tax on shisha they r trying to scare people from it. My relos should of been dead years ago if this was true.

Of note, the cynical comments did not generally tag other people, compared with other categories of comments.

### Unclear

There were a significant number of comments which were classified as unclear as to whether they accepted or rejected the campaign message. Of these, there were three specific comment themes that recurred throughout the data. The first involved comments that simply had ‘No thanks’ (n=71; 1.4%), which did not indicate whether the commenters were being sarcastic or not, or whether they were saying ‘no thanks’ to shisha, or ‘no thanks’ to the campaign video. The second subcategory was comments where people were asking genuine questions (n=17; 0.3%), demonstrating they were interested and engaged with the topic, but that they were undecided whether to accept or reject the campaign message. These could either be questions to a friend asking for their thoughts, for example:

- [name] what do ya think
- [name] true or bs?

or genuine questions to the organization, for example:

- What about the herbal, non-tobacco variety? Surely nothing wrong with that?
- [name] how do they make the comparison?

Another theme that was present in some of the comments was that the commenter felt the campaign was either a personal or cultural attack (n=35; 0.7%). Some people felt that the campaign video, or possibly after being tagged on the video by friends, was personally attacking them, for example:

- [name] [name] I personally feel attacked
- [name] I feel like this ad is a personal attack
- [name] I feel personally attacked by the government.

Others implied that the campaign was an attack on a specific culture, for example:

- How racist is this but [name]
- [name] [name] this is a direct attack on my culture and identity
- [name] the health department is cracking down on culture.

Finally, there was a proportion of comments (n=742; 14.9%) which were clearly relevant to the campaign topic or message, but it was not possible to interpret the meaning of these comments as to whether the commenter accepted or rejected the campaign message.

## DISCUSSION

This study’s analysis of social media comments is a valuable component to evaluating the *Shisha No Thanks* project, as it provides insight into people’s response to the campaign message. Based on the dataset of 4990 comments, 9.1% expressed clear acceptance of the campaign message, with 2.8% of comments indicating priming steps of behavioral change of the commenter expressing intention to stop smoking shisha, or asking a friend to stop smoking shisha. In contrast, 22.9% of comments rejected the campaign message, with the majority of those being people laughing at the campaign video or expressing pro-shisha sentiments.

This study demonstrates the value of thematically analyzing social media comments. The majority of public health campaign evaluations use only process evaluation measures of social media metrics (such as reach, impressions or likes)^1^ or impact evaluation measures of changes in attitudes and behaviours^5^. Both those aspects of evaluation are important, but have their limitations, primarily in not illustrating what happens between the dissemination and reach of the campaign, and the actual intended campaign outcomes. Social media comments can reveal this intermediary step and indicate whether the campaign message has actually ‘landed’, and how it has been understood and received by the target audience.

This study demonstrates that in the *Shisha No Thanks* campaign, a small but important proportion of people who viewed the video understood, accepted and took up the campaign message, by either saying they themselves would stop smoking shisha, or by asking a friend to stop smoking shisha. Further, the comments provide insight into aspects of the campaign that resonated most, for example, the message that ‘45 min (of shisha smoking) equals 100 cigarettes’.

Conversely, analyzing the Facebook comments also provides insights into the proportion of people who, despite viewing and engaging with the video, did not seem to take up the campaign message. This demonstrates that process indicators such as video views or engagement metrics alone do not tell the full story. The comments also provided insight into some of the reasons why people did not accept the campaign message^33^, which is particularly important given the large proportion of comments in this group. One of the common themes was skepticism toward the motives behind the campaign, with commenters cynically implying that the ‘government’ did not want people to smoke shisha because they would receive less tax revenue than if shisha smokers switched to cigarettes. This suggests one reason for the low acceptance of the campaign messages is the view of mistrust and wariness towards the ‘messenger’ (a government agency) among the audience. Another potential reason for the low acceptance of the campaign message is the strong social and cultural ties that shisha has among groups^21,22^, and the general social acceptability of shisha smoking^19^. In considering comments that rejected the campaign, it is worthwhile to note that research into tobacco control campaigns has found that messages that portray health consequences of smoking and evoke strong negative emotions are actually effective^34^, and therefore a strong negative reaction may not necessarily be an indication of ineffectiveness of the campaign.

Finally, analyzing Facebook comments helped the project team to understand other potential unintended effects of the campaign, including the perception that the campaign attacks a community’s cultural practice. There was substantial concern about this during development of the campaign, but the very small proportion of comments that expressed this sentiment (n=35; 0.70%) suggests that the video and broader campaign were culturally sensitive. This is an important finding that shows that a co-design approach can help manage the cultural sensitivities of campaigns on this issue.

### Conceptualizing social media comments in campaign evaluations

**Figure 2:**
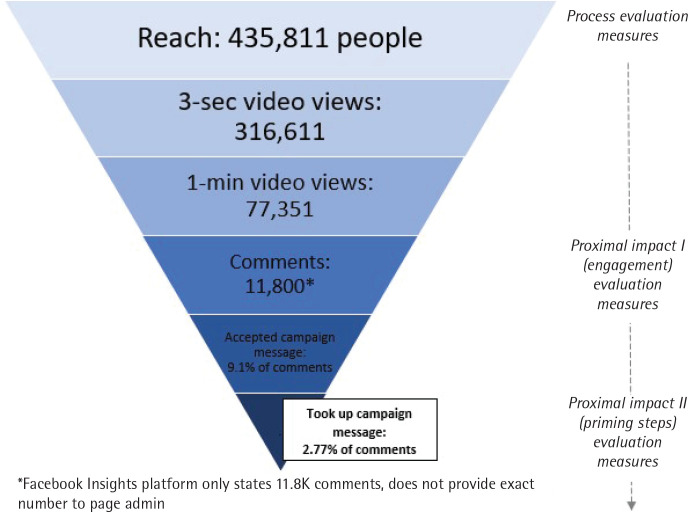
Levels of engagement with campaign video

Social media comments can be seen as a more nuanced form of engagement, than the more rudimentary metrics of ‘likes’ and ‘reactions’, as they provide more insight into the sentiment of the individual towards the campaign, and as demonstrated in this study, can even indicate intentions to change behavior (priming steps). Building on the framework of other campaign evaluation models^5,35,36^, this evaluation study shows that incorporating social media comments into the evaluation process through content analysis could provide an indicative proximal impact evaluation measure of intention to change behavior (priming steps). Each level of evaluation metric shows diminishing numbers, but increased participation in the campaign, and progress towards the desired campaign outcomes (Figure 2).

### Strengths and limitations

A strength of this study is the involvement of cultural support workers in the analysis process of the study. Their involvement ensured appropriate cultural and linguistic interpretation of the comments, and is in keeping with the co-design principles of the project, which aimed to involve community members throughout the project, including the evaluation. The involvement of the *Shisha No Thanks* project officer, and a staff member from WSLHD is also seen as a strength of the study, as they were able to provide helpful context to some of the references in the comments, as they had regular interactions and conversations with the video’s audience. An additional strength of this study is the inclusion of emojis in the comment analysis. During the analysis process, the study team recognized that the emoji pictures that were provided, carried a lot of meaning and provided key information in understanding the tone, and therefore category, of the comment. For example, this comment was categorized as accepting the campaign message: ‘[name] for you guys’; whereas this comment was categorized as rejecting the campaign message: ‘[name] for u 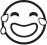
<face with tears of joy>’, as the emoji changed the tone from serious to joking. In addition, many comments only consisted of emojis and tagged names, with no other text (e.g. ‘[name]<face with tears of joy>
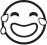
<face with tears of joy>’; ‘[name] 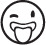
<winking face with tongue>
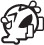
<rolling on the floor laughing>’; ‘[name]
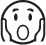
<face screaming in fear>’). In these instances, the emojis provided the whole meaning of the comment.

A limitation of this study is that we were only able to export part of the total number of comments posted to this Facebook post (slightly less than half of total comments), due to the Facebook Graph API limits. It is not clear from the information provided by Facebook what rules are used in selecting which comments get exported, such as whether they are the most recent comments, the comments with the most engagement, or a random sample of comments. In addition, it is not apparent why 9 comments were missing in the extraction data (as only 4991 comments were returned). While this is not ideal, this represented only a very small proportion (0.2%) of the total number of comments we reviewed. Another limitation of this study is that we did not have information about the demographics of the people who posted comments on this video on WSLHD’s Facebook page, and so there is no way to identify whether the people who commented on the video were from the project’s target audience of young people of Arabic-speaking background. However, Meta (Facebook’s parent company) has reported that in Australia, 43.4% of the combined Facebook, Instagram and Messenger advertising audience is in the 18–35 years age group (the target audience of this project)^37^. In addition, some of the comments posted to the video included individual Arabic words, which suggests that at least some of the commenters were of Arabic-speaking background. Furthermore, we acknowledge that people who leave comments on social media posts are more likely to be people who have a strong opinion on the topic, which may limit the generalizability of these findings to the wider video audience.

There were significant challenges in interpreting the Facebook comments, which is reflected in the large proportion categorized as ‘Unclear’ as to whether they accepted the campaign message (n=1089; 21.82%). This is due to the difficulty in interpreting tone in written comments (i.e. whether the commenter is being serious or sarcastic), the lack of context of the comments and having no understanding of the relationship between the commenter and the person they have tagged, and the specific culture that is embedded in social media comments. Specifically, there were examples where the commenter believed the campaign message or saw its personal relevance, but did not take it seriously, for example: ‘
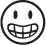
<astonished face> + 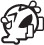
<rolling on floor laughing>’, or ‘[name][name] cut that sh.t out yeah 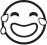
<face with tears of joy>’.

## CONCLUSIONS

This study is one of the first to provide insights into how messages that raise awareness of the harms of shisha use are processed by people on social media. Campaigns such as the *Shisha No Thanks* project are important in providing evidence-based messages about shisha smoking, raising awareness of the harms of shisha, and countering the large volume of pro-shisha content on social media^11,12^.

## Data Availability

The data supporting this research are available from the authors on reasonable request.
